# A Method for the Analysis of AP Foot Convexity: Insights into Smooth Muscle Biophysics

**DOI:** 10.3389/fbioe.2017.00064

**Published:** 2017-10-26

**Authors:** Shailesh Appukuttan, Mithun Padmakumar, Keith L. Brain, Rohit Manchanda

**Affiliations:** ^1^Computational Neurophysiology Lab, Department of Biosciences and Bioengineering, Indian Institute of Technology Bombay, Mumbai, India; ^2^College of Medical and Dental Sciences, Institute of Clinical Sciences, University of Birmingham, Birmingham, United Kingdom

**Keywords:** action potential, parasympathetic neurotransmission, convexity, quantification, propagation

## Abstract

Action potential (AP) profiles vary based on the cell type, with cells of the same type typically producing APs with similar shapes. But in certain syncytial tissues, such as the smooth muscle of the urinary bladder wall, even a single cell is known to exhibit APs with diverse profiles. The origin of this diversity is not currently understood, but is often attributed to factors such as syncytial interactions and the spatial distribution of parasympathetic nerve terminals. Thus, the profile of an action potential is determined by the inherent properties of the cell and influenced by its biophysical environment. The analysis of an AP profile, therefore, holds potential for constructing a biophysical picture of the cellular environment. An important feature of any AP is its depolarization to threshold, termed the AP foot, which holds information about the origin of the AP. Currently, there exists no established technique for the quantification of the AP foot. In this study, we explore several possible approaches for this quantification, namely, exponential fitting, evaluation of the radius of curvature, triangulation altitude, and various area based methods. We have also proposed a modified area-based approach (C_X,Y_) which quantifies foot convexity as the area between the AP foot and a predefined line. We assess the robustness of the individual approaches over a wide variety of signals, mimicking AP diversity. The proposed (C_X,Y_) method is demonstrated to be superior to the other approaches, and we demonstrate its application on experimentally recorded AP profiles. The study reveals how the quantification of the AP foot could be related to the nature of the underlying synaptic activity and help shed light on biophysical features such as the density of innervation, proximity of varicosities, size of the syncytium, or the strength of intercellular coupling within the syncytium. The work presented here is directed toward exploring these aspects, with further potential toward clinical electrodiagnostics by providing a better understanding of whole-organ biophysics.

## 1 Introduction

**Figure 1:**
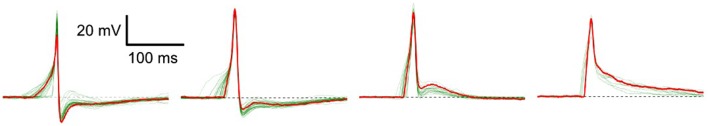
Diversity in action potentials observed in detrusor smooth muscle cells. The green traces in each panel represent different instances of APs having a similar profile. A typical AP shape belonging to each group is highlighted in red.

Excitable cells are characterized by their ability to produce action potentials (APs). Typically, cells of the same tissue, or specific region of tissue, exhibit a common AP profile characteristic of that cell type. But in certain syncytial tissues, such as the smooth muscle layer of the mouse urinary bladder wall (called the detrusor), individual cells are known to exhibit diversity in AP shapes (Meng et al., 2008). These APs do not exhibit any pattern of changes in shape, rather the variation of shape from any given AP to the succeeding ones is seemingly random in nature. Some of the diverse shapes recorded intracellularly—as described in Section 2.4—from detrusor smooth muscle cells (DSMCs) are shown in Figure 1. Neither their origin nor the physiological role of this diversity is currently understood, but has often been attributed to syncytial interactions and the spatially distributed pattern of parasympathetic innervation (Manchanda, 1995). It may be assumed that changes in the underlying cellular or tissue features influence the shape of the produced AP. These changes can affect the cellular biophysics and result in a variation of the AP profile (Appukuttan et al., 2015a, 2017a). As the APs holds the ability to generate phasic and tonic contractions of the DSM tissue, their analysis of AP shapes holds potential to help identify and diagnose pathological conditions.

**Figure 2:**
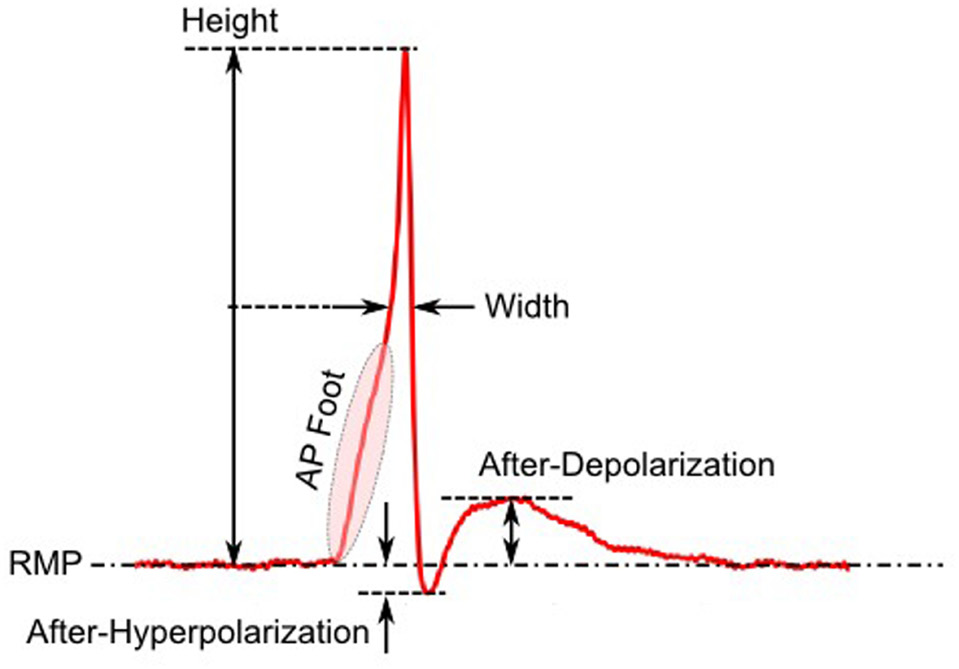
Parameters employed for quantification of AP profile.

**Figure 3:**
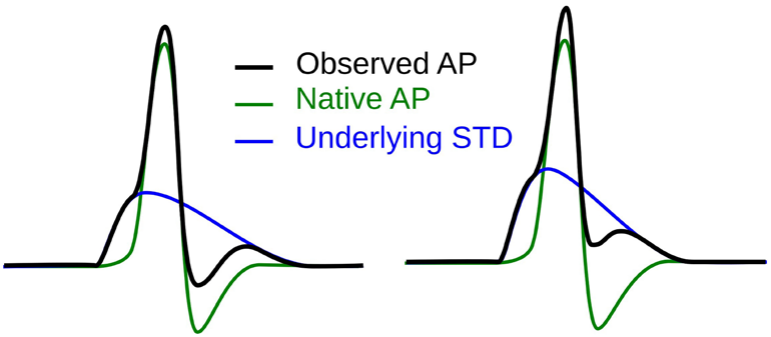
Hypothesis for the generation of sAPs involving the variable superposition of an underlying STD and a native AP profile.

The first step toward interpreting the observed AP diversity in the detrusor would involve the characterization of AP profiles to enable their comparison and analysis. Historically, APs are most often characterized in terms of their height and width (full width at half maximum or FWHM) as shown in Figure 2. Other parameters used to describe APs include overshoot, after-hyperpolarization (AHP) and after-depolarization (ADP) (Bean, 2007). An important feature of any AP is its rising phase, which can be divided into two parts: (i) the passive depolarization from the resting state (resting membrane potential or RMP) to a threshold value, and (ii) the rapid, active depolarization beyond the threshold to the peak of the AP, propelled by voltage-gated channels. The former region, termed the foot of the AP, holds information about the biochemical and spatial origin of the AP. For example, in skeletal muscle fibers, it has been shown that APs produced close to the end-plate have a convex-upward rise to peak. With increasing distance from the end-plate, this gradually changes from convex-upward to a concave-upward rise to peak (Fatt and Katz, 1951). Thus, from the AP profile, it can be determined whether it was recorded close to the site of synaptic activity or far from it. Similar observations have been reported in case of neurons during the propagation of AP away from the soma (Magee and Carruth, 1999). For simplicity, we shall refer to convex-upward and concave-upward rise to AP peak as convex and concave AP foot, respectively. In the case of the detrusor, it has been hypothesized that the diversity in action potential shapes is owing to the variable superposition of spontaneous transient depolarizations (STDs; similar to miniature end-plate potentials in skeletal muscle) and an unmodulated AP profile (Padmakumar et al., 2012), as illustrated in Figure 3. Spontaneous neurotransmitter release from parasympathetic varicosities produces STDs in DSMCs, which on crossing the AP threshold of the cell, elicits an AP. The STD, being a passive signal, decays with increasing distance from the varicosities, as in Fatt and Katz (1951). The active AP signal, on the other hand, has the capacity to propagate, often without attenuation. The presence of a STD underlying an AP is reflected in the convexity of the AP foot. The propagating APs which travel significant distances from the source will have no underlying STD component (Appukuttan et al., 2015b), and hence would exhibit a concave foot. Such AP profiles are denoted as “native” APs in the current study. The extent of STD content is reflected in the degree of convexity of the AP foot.

The quantitative analysis of AP foot can provide leads concerning the nature of an AP, e.g., the location of the point at which AP is observed vis-à-vis site of neurotransmission. This can help construct a “biophysical picture” of pattern and density of parasympathetic innervation in the vicinity of a given recording point. In the past, similar profiling of electrical activity has helped interpret the nature of autonomic neurotransmission in tissues such as the vas deferens and arterioles (Hirst and Neild, 1978; Blakeley and Cunnane, 1979; Cunnane and Stjärne, 1984; Brock and Cunnane, 1988). In past studies in mouse DSM, the differences in the convexity of the foot of the AP were observed to distinguish between neurogenic and myogenic APs (Meng et al., 2008; Young et al., 2008).

Currently, there is no well-defined method for the quantification of the AP foot convexity. The ideal technique that quantifies the AP foot should be able to provide us an idea about the underlying passive signals which brings the membrane potential to threshold and gives rise to the AP. In the present work, we have attempted to explore various approaches to measure AP foot convexity. It could be demonstrated that all these methods suffer from certain shortcomings which restrict their utility for our purpose. We have thus designed a novel approach which overcomes these drawbacks and was found to be appropriate for the quantification of AP foot convexity. The various approaches have been evaluated and compared using a set of test data mimicking AP diversity, where the actual convexity trends were known. Finally, we demonstrate how the proposed technique performs on experimentally recorded AP profiles and discuss its possible utility in interpreting electrophysiological activity in health and disease.

## 2 Methods

This section is divided into three parts, dealing with the synthesis of the test data, the evaluation criteria, and the description of the various quantification methods explored, including our proposed technique.

### 2.1 Synthesis of Test Data

A data set consisting of diverse AP shapes was required for evaluating the various quantification approaches. A prerequisite was foreknowledge about the convexity trends of the data set, against which the approaches could be compared. Experimental data could be not used for the evaluation of the proposed techniques because prior knowledge of their extent of AP foot convexities was unavailable. It was thus necessary to generate the test data computationally.

Here, one of the possibilities was to obtain AP profiles from simulations on a syncytial model (Appukuttan et al., 2015a) by recording at each model cell. But this approach had a couple of drawbacks. First, even though syncytial spread of APs leads to change in the AP shape (Appukuttan et al., 2015a), the number of widely differing AP profiles so obtained would be limited. This owes to the fact that the syncytium is homogeneous and well coupled, and the variabilities in DSM bundle sizes and the neurotransmitter release profiles were not implemented in the available model, restricting the AP shape diversity observable from the model cells. The second drawback was the inability to accurately predict the foot convexity trends for varying superpositions of the passively spreading synaptic stimulus and the AP. This is because when the signals are recorded from the syncytium, the underlying STD undergoes multiparametric variations such as its amplitude, time course, and the latency with respect to the AP. Further, the inability to explicitly set the AP threshold in models, such as in the HH model, proved another hindrance and limited the range of STD amplitudes that could be availed.

**Figure 4:**
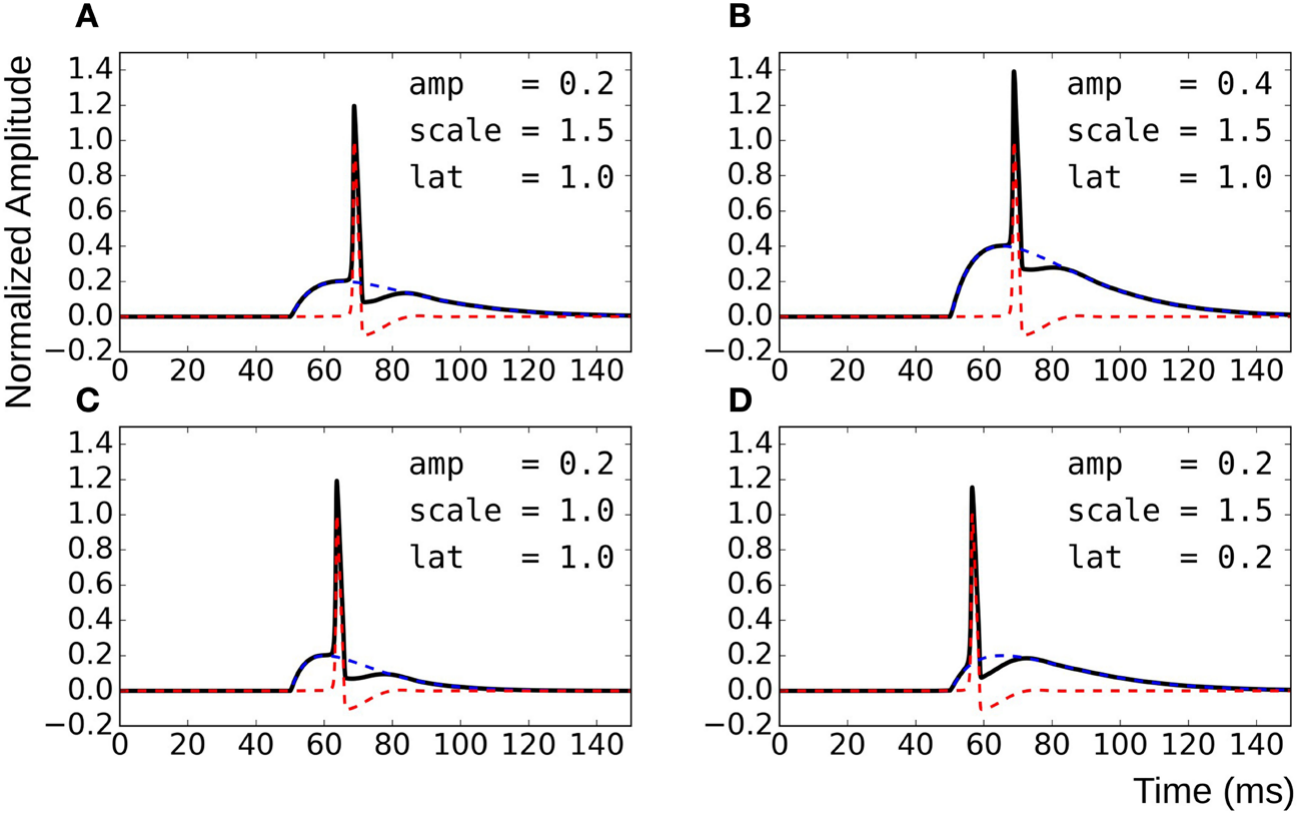
Method for generating test data by varying superposition of a modulated STD with an AP profile. **(A)** reference panel, **(B)** change in STD amplitude, **(C)** change in STD time course, and **(D)** change in AP latency.

To overcome these shortcomings, the test data were synthesized by direct linear superposition of two template signals: (i) an AP profile with a concave foot and (ii) a STD representing membrane response to synaptic stimulus. These template signals were obtained from simulations on the NEURON environment, a compartmental modeling platform (Hines and Carnevale, 2001), using the syncytial smooth muscle model (Appukuttan et al., 2015a). In order to obtain the AP template, all cells in the syncytium were endowed with Hodgkin–Huxley (HH) channels and the stimulus, implemented via an alpha function mimicking an STD profile (Purves, 1976; Bennett et al., 1993), applied at the centroid cell. The AP recorded at the vertex cell had the most concave foot by virtue of being farthest from the site of stimulation, and thus was chosen as the AP template. The amplitude was normalized such that RMP was at 0 and the peak had a value of +1. The STD template was obtained from the centroid cell when all cells in the syncytium were rendered purely passive. Experimental studies have shown STDs in smooth muscle to be of varying amplitudes and time courses (Manchanda, 1995). The test data was thus generated using varying levels of superposition of the AP template with modulations of the STD template. This was achieved by varying three principal parameters: (i) the amplitude of the STD (*amp*), (ii) the time course of the STD (*scale*), and (iii) the latency of AP onset from STD onset (*lat*). Parameter *amp* was relative to the normalized AP template; a *scale* value of 1 represented the original STD time course, with values lesser and greater than one indicating shortening and elongation, respectively, in time domain by that factor; *lat* of 0 and 1 indicate alignment of AP onset with STD onset and with STD peak, respectively. The effects of varying each of the parameters are illustrated in Figure 4. Different test data sets were created by varying a single parameter, while keeping the other two constant. For every data set thus generated, it was possible to predict their intrinsic foot convexity trends, as explained in the following section. This information was used to evaluate and compare the robustness of various quantification techniques described ahead. The details of the generated data sets are provided in the Results section.

### 2.2 Evaluation Criteria

Knowledge of the origin of the test data sets allows us to make certain assertions, such as the relative ordering of signals with respect to their foot convexities. Such orderings are possible within each data set individually, but cannot be ascertained for the combination of the data sets together. This is because the convexity ranges would overlap between the sets, and the rank of individual signals in the combined set cannot be predicted easily. It can be observed from Figure 4 that the convexity of the AP foot increases with each of the parameters *amp, scale*, and *lat*. Thus, an increase in the amplitude and time course of the STD would result in an AP with a more convex foot, and this would be further heightened with a longer latency of AP onset.

Another assertion that could be made with regard to the test data is the relation between the AP foot convexity and the magnitude of ADP. The ADP is defined as a membrane depolarization observed after the AP peak, but before the membrane potential settles back to its RMP. Here, the ADP is evaluated as the difference between the first local maximum, following the AP peak, and the RMP. The nature of the relation between convexity and ADP varies depending on the parameter that is changed. Here, an increase in the amplitude and time course of the STD leads to an increase in the ADP of the AP profile, whereas a longer latency of AP onset would reduce the extent of this ADP. Thus, a positive positive correlation is expected between each of *amp* and *scale* with ADP, and a negative correlation between ADP and *lat*. As the increments in convexity in each data set are not strictly linear, but the intrinsic ordering is known, the Spearman’s rank correlation coefficient (*ρ*) was used to test the correlation between two variables.

We employ the above two ground truths to evaluate the various quantification approaches described in the following section. Before proceeding further, it would be useful to formally demarcate the AP foot region. In our present study, the AP onset for each signal in the test data is predetermined by the instance of synaptic activation (50 ms from the start of the simulation). This is set as the onset of the AP foot. The end of the foot (EOF) is determined differently for convex and concave rise to AP. For each signal, we attempt to locate an inflection point between the foot onset and the AP peak. In case of APs with convex feet, such a point would exist discernibly and is chosen as the EOF. But in the case of APs with concave feet, discernible inflection points would be absent and thus, as an alternative, the EOF is set as the instant where the AP has the maximum rising slope. Through simulations it has been verified that this corresponds to the time instance at which the capacitive current, arising from local circuit pathways, attains its peak. Also, it was observed that this occurs 0.1 ms after the initiation of the regenerative mechanism of Na^+^ channels, leading to rapid depolarization beyond the threshold.

Finally, we demonstrate the application of the proposed method in quantifying the foot convexities in a set of experimentally recorded APs. The experimental recordings are different from the simulated signals in three major aspects: (1) there is significant noise content, (2) there exists much greater diversity in AP shapes, and (3) the exact location of the onset of the signal and the EOF cannot be easily identified. These differences demand certain amendments to the signal processing techniques employed in analyzing the simulated signals, as discussed by Padmakumar et al. (2016). Once the AP foot is isolated, the convexity detection algorithms can be directly applied on the experimental signals, as in the case of test data sets. However, the noise content in the foot might affect the efficiency of some techniques that involve the first and second derivatives. This problem was overcome by low pass filtering (cut off frequency = 40 Hz) of the signal foot before applying the technique.

### 2.3 Convexity Quantification Approaches

#### 2.3.1 Radius of Curvature

The radius of curvature (RoC), *r_i_*, at the *i*^th^ instant of a signal *s* is given by the following equation (Kreyszig, 1991):

(1) $$r_{i}=\frac{{\left\{1+{\left(s_{i}^{\prime }\right)}^{2}\right\}}^{\frac{3}{2}}}{s_{i}^{\prime \prime }}$$

**Figure 5:**
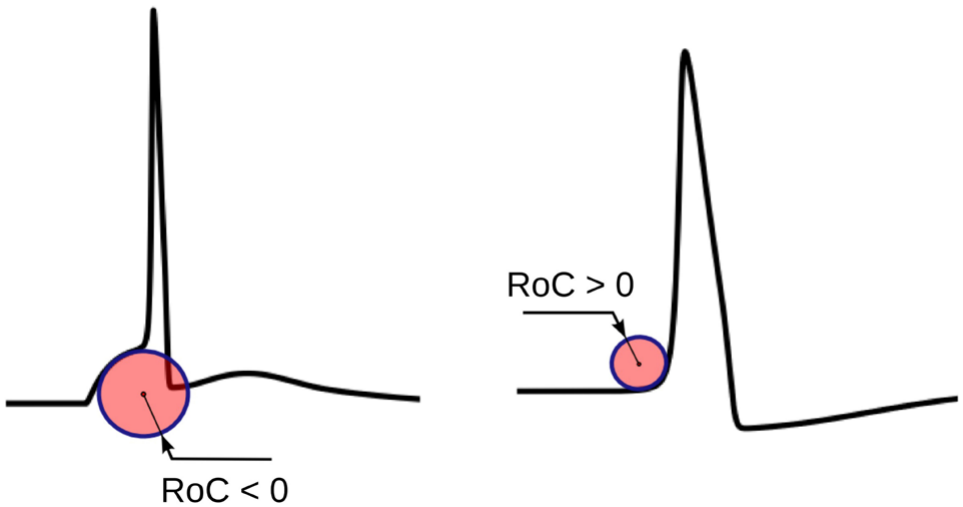
The physical significance of the radius of curvature (RoC). The RoC values measured at convex foot is negative and that in concave foot is positive. These signs are flipped to match the convention followed in other techniques.

The physical significance of *r_i_* is shown in Figure 5. *r_i_* evaluated using equation (1) would yield negative values for convex-foot APs and positive for concave-foot APs. To ensure that the sign is consistent with the convention followed in other techniques described below, we define *ř_i_* as the negation of *r_i_*:

(2) $${ř}_{i}=-r_{i}$$

and employ this for the quantification of convexity. The value of *ř_i_* is used to quantify the convexity of the foot in three different ways, as follows:

1. *Minimum radius*: the minimum absolute value among the radii measured along the foot is taken as the measure of convexity, while maintaining its sign.
2. *Mean radius*: the convexity measure is obtained by averaging the instantaneous radii along the entire AP foot.
3. *Total radius*: the radius measured at each instant along the foot is added to obtain a measure of the convexity. This method differs from the mean radius in its ability to account for the foot width, i.e., if the foot is wider, the convexity measure becomes larger for convex signals, and smaller in case of concave signals.

Here, we shall denote these convexity measures by C_rad_. Evaluation of the mean and total cases require certain adjustments to eliminate errors arising from extremely low curvature regions (~0), i.e., extremely high |*ř_i_*|, and can be managed by setting a ceiling for |*ř_i_*|.

#### 2.3.2 Exponential Fit

**Figure 6:**
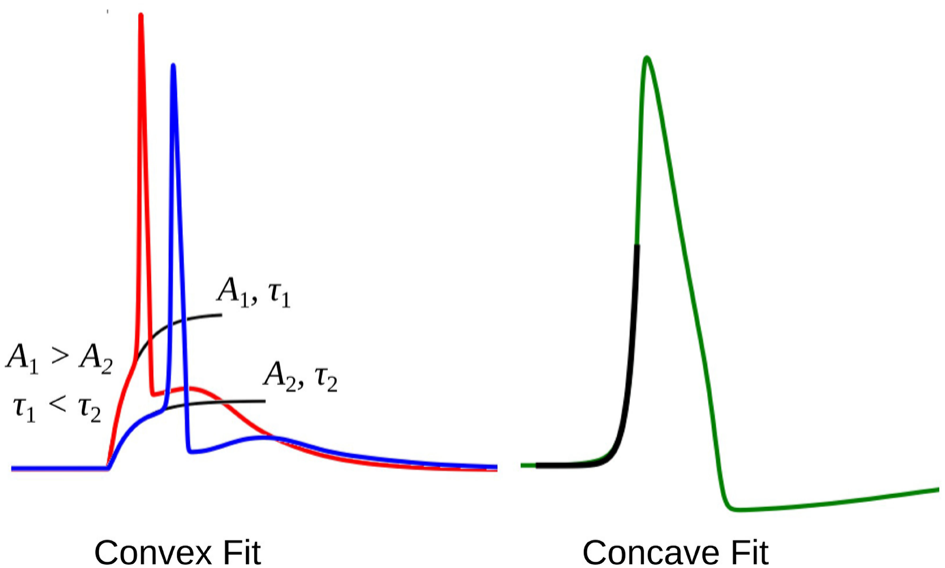
The exponential fit for two convex footed APs (left) and one concave footed AP (right). The comparison of the fitting parameters corresponding to the two convex foots are also shown. See text for details.

The rising part of the STD, which constitutes the foot of convex APs, can be approximated as a saturating rising exponential having the following form:

(3) $$v=A(1-e^{-t/\tau })$$

where *v* is the membrane potential and *t* the time instant relative to the AP onset. The parameters for the exponential fit, namely the time constant *τ* and the scaling factor *A*, could be used as a measure of convexity, as shown in Figure 6. Cable theory predicts that the passive signals upon transmission through a cable-like structure would widen in its time course, resulting in an increase in the time constant *τ* (Jack et al., 1975; Bywater and Taylor, 1980). Also, the amplitude of the STD would fall with propagation, resulting in reduced amplitude of the scaling factor *A*. In our test data, since we have considered both variations in the underlying STD amplitude and also its time course, the quantification of the convexity would need to consider both the factors, *A* and *τ*.

In case of concave AP foot, equation (3) cannot be used, and is better approximated by a non-saturating exponential rise of the following form:

(4) $$v=A(e^{t/\tau }-1)$$

During quantification, we denote the time constant of the exponential fits using equation (4) as negative, in order to differentiate it from the convex rise. When analyzing an unknown signal, exponential fits are determined using both equations (3) and (4), and the one with the least root of sum of squares of errors is selected. We shall denote these convexity values by C_exp_.

#### 2.3.3 Triangulation Altitude

**Figure 7:**
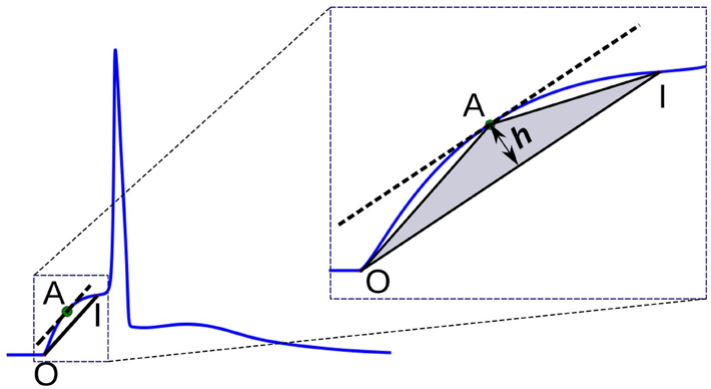
Convexity evaluation using triangulation altitude method. O and I represent the onset and end of the foot, respectively. Point A represents the apex of the triangle at which the tangent of the foot is parallel to the base OI of the triangle. *h* is the altitude used as a measure of foot convexity.

In this method, a triangle is formed using the onset of the AP foot “O,” the end of the foot “I,” and a point “A” chosen on the AP foot such that the altitude of the triangle OAI from the base OI is maximized. It could be noted that this condition is satisfied when “A” is located at a point where the tangent at “A” is parallel to the base OI. The altitude *h* provides a measure of the convexity of the foot. This approach is illustrated in Figure 7. For APs with concave foot, the orientation of the triangle would be inverted over the base OI, and the altitude so measured is assigned a negative sign. We shall denote the convexities measured using this approach as C_alt_.

#### 2.3.4 Area Based Approaches

**Figure 8:**
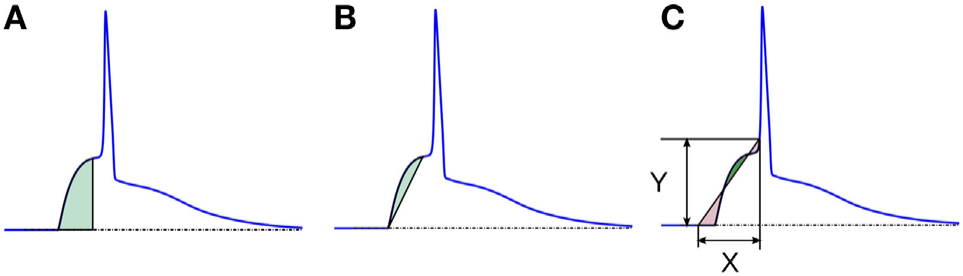
Different schemes of area based convexity measures: **(A)** area under the foot, **(B)** area between the foot and the line joining the foot onset and EOF, and **(C)** area between the foot and a predefined line having width X and height Y. The areas shaded with green are measured positive and those shaded red are measured negative. For details, see text.

It is possible to quantify the convexity of the foot by evaluating the area associated with the AP foot. This area could be measured in multiple ways, as follows:

1. *Area under the AP foot: here*, the area measured is always positive, as the foot never goes below the resting membrane potential (RMP). The larger the value, higher is the convexity. This method is illustrated in Figure 8A. We denote these convexities as C_area_.
2. *Area between the AP foot and the line joining the foot onset and EOF: here*, the reference line is changed from the X-axis (in above case) to a line joining the two ends of the foot (see Figure 8B). The regions where the curve is above the line are considered as positive, and below it as negative. We shall denote this convexity measure as C_line_. It can be noted that the length and the slope of the line will vary between signals.
3. *Area between the AP foot and a predefined line: this* is illustrated in Figure 8C. One end of the line is located on the AP at the instant where the AP signal crosses a predefined depolarization level Y above the RMP. The other end of the line is set X ms away, toward the foot onset, with ordinate equal to the RMP. The area between the curve and this line is then evaluated, with regions above the line being considered positive, and the area below it as negative. The convexity measure is denoted as C_X,Y_, representing the measure of Convexity (C) determined for a depolarization of Y over a time period of X ms. In this approach, the length and the slope of the line remains the same across all signals for a given pair of X and Y. While demonstrating this approach on the test data set, we shall evaluate C_20,0.6_, i.e., C_X,Y_ with X = 20 and Y = 0.6. Later we discuss how this method behaves for different values of X and Y, and how to select appropriate values for these parameters.

### 2.4 Electrophysiological Recordings

#### 2.4.1 Ethics Statement

The Institutional Animal Care and Use Committee (IACUC) covering the Department of Pharmacology, University of Oxford, approved and had oversight of all animal experiments. All experiments were carried out during or before 2009 and were hence approved under Animals (Scientific Procedures) Act 1986, but are also consistent with both UK Animals (Scientific Procedures) Act (2013) and European Communities Council Directive 2010/63/EU.

#### 2.4.2 Experimental Protocol

Mice of the C57BL/6 strain, of either gender, weighing 18–30 g, were used to collect electrophysiological recordings. They were killed by head concussion, followed by cervical dislocation. Efforts were undertaken to minimize the number of animals used in the study, and to reduce their suffering. The urinary bladder was removed with the connective tissue surrounding the bladder removed, while the urothelium was left intact. The ventral wall of the bladder was opened longitudinally from the bladder neck (posterior) to the top of the dome (anterior). Tissue strips, which contained a few bundles of smooth muscle, 3–4 mm long and 1–2 mm wide, were dissected. Strips were pinned out on a Sylgard-lined plate at the bottom of a shallow chamber (volume, approximately 1 ml). This was mounted on the stage of an upright microscope. Preparations were superfused with physiological saline solution (PSS) (composition, mM: NaCl, 120; KCl, 5.9; MgCl_2_, 1.2; CaCl_2_ 2.5; NaHCO_3_, 15.5; NaH_2_PO_4_, 1.2, and glucose, 11.5; gassed with 95% O_2_ and 5% CO_2_) warmed to 35°C, at a constant flow rate of 100 ml/h, and maintaining a pH of 7.2–7.3 (Hashitani and Brading, 2003).

Preparations, once pinned, were allowed to equilibrate for at least 30 min before initiating electrophysiological recording. Individual bladder smooth muscle cells in muscle bundles were impaled with glass capillary microelectrodes, filled with 0.5 M KCl, with a tip resistance of 100–300 MΩ. Changes in the membrane potential were recorded using a high input impedance amplifier (Axoclamp-2B, Axon Instruments, Inc., Sunnyvale, CA, USA), and were digitized using PowerLab/4SP (AD Instruments, Chalgrove, UK) at either 1 or 4 kHz, and stored on computer for later analysis.

## 3 Results

### 3.1 The Test Data Sets

Three independent data sets (Dataset1, Dataset2, Dataset3) were created; one for variations in each of the three parameters *amp, scale*, and *lat* described in Section 2.1, while the remaining two were kept constant. Each data set was designed to contain 25 different AP profiles. As these data sets lack the presence of concave footed APs, an additional data set (Dataset4) was generated comprising a near proportionate mix of APs with concave and convex AP feet (concave: 11, convex: 14). Table 1 describes the ranges of parameter values employed in generating the test data. As explained in Section 2.2, the level of foot convexity increases with an increase in the varying parameter in each data set.

**Table 1:** Variations in parameter values to generate test data sets.

Data set	Amplitude	Scale	Latency
Dataset1	0.08 to 0.5	1.5	1.0
Dataset2	0.2	0.2 to 1.4	1.0
Dataset3	0.2	1.0	0.0 to 1.5
Dataset4	0.2	1.0	−0.2 to 0.25

### 3.2 Comparison of Convexity Measurement Algorithms

For each of the data sets, the AP foot convexity was evaluated using the algorithms described in Section 2.3. As established in Section 2.2, a strong positive correlation was expected between the parameter being varied in the data set (*amp, scale, lat*, and *lat* for Datasets 1, 2, 3, and 4, respectively) and the measured convexity. The Spearman’s rank correlation values were evaluated between the measured convexities and the parameter varied in each of the data sets, and the results are tabulated in Table 2. It can be observed that the proposed method, C_X,Y_, yields the best result by producing the expected correlation of + 1 for all data sets. Other area based methods C*_area_* and C*_line_* also perform well in all data sets except Dataset4 (correlation = 0.83 for both). Since C*_area_* is a special case of C*_line_* where the ordinate of the reference line is set to RMP, it was reasonable that a similar trend should be obtained from both methods. The uniqueness of Dataset4 was in its composition of a mix of AP profiles with varying extents of AP foot concavity and convexity, while the other data sets predominantly consisted of AP profiles having convex AP feet, with at most a single concave AP profile in each set. On closer examination, it was noted that the nature of convexity trends shown by C*_area_* and C*_line_* were opposite for convex and concave feet. They produce a positive correlation with *lat* for convex feet, and a negative correlation for concave feet, causing the overall correlation value to drop for Dataset4, as seen in Table 2.

**Table 2:** Spearman’s rank correlation between the convexity measures obtained via the different quantification approaches and the parameter varied for each data set.

Approach	Dataset			
	1	2	3	4
C_rad:min_	−1.00	1.00	0.77	0.86
C_rad:mean_	−1.00	0.99	1.00	0.82
C_rad:total_	−1.00	1.00	1.00	0.83
C_alt_	1.00	−1.00	1.00	0.83
C_exp:A_	1.00	1.00	−0.77	0.73
C_exp:_*_τ_*	−1.00	1.00	−0.37	0.21
C_area_	1.00	1.00	1.00	0.83
C_line_	1.00	1.00	1.00	0.83
C_20,0.6_	1.00	1.00	1.00	1.00

The triangulation altitude, C*_alt_* performs well for Datasets 1 and 3. However, it presents the same issue as that faced by the C*_area_* and C*_line_* methods; i.e., the opposing sub-trends within Dataset4. It is interesting to note that C*_alt_* generated a strong negative correlation (−1.0) for Dataset2, where the parameter *scale* is varied. This could be explained by the fact that C*_alt_* measures the depth of the curvature formed by the AP foot by the STD, which drops with increase in *scale* parameter.

The algorithms based on the radius of curvature produce maximum correlations for Datasets 1 and 2 but with opposite signs. The variation in *amp* produced a negative correlation with the convexity values measured by radius of curvature. This can be attributed to the increase in the foot curvature when the foot height is enhanced, while its width remains unchanged; resulting in a decrease in the radius of curvature, and thus the negative correlation. Increase in *scale*, however, causes an increase in the measured radius of curvature. For Dataset3, the reduction in the correlation obtained by C*_rad:min_* was caused by the dual trend of the C*_rad:min_* values obtained before and after *lat* = 1. The radius of curvature increases monotonically and attains maximum value at *lat* = 1 and then stays roughly constant, with a negligible decrement in RoC as the *lat* parameter increases further. The reason for such behavior lies in the curvature profile of the STD. At *lat* = 1, the AP onset is aligned to the STD peak. However, the change of trend seen in C*_rad:min_* was absent for the C*_rad:mean_* and C*_rad:total_* values because the instantaneous RoC values measured at the initial part of the foot compensates for the decrement at the end points. For Dataset4, as expected, the curvature based methods could not produce similar trends for convex and concave footed APs and thus have lower correlation values.

While evaluating the convexity using C*_exp_*, it is expected that the scaling factor *A* would solely depend upon the STD amplitude (*amp*). Hence, maximum variation in *A* occurs during the evaluation of Dataset1, where the parameter *amp* is varied. For other data sets, the variation in *A* is negligible. Similarly, the time constant *τ* depends on the STD rising phase which is varied using the *scale* parameter in Dataset2. For other data sets, the variation in *τ* is negligible. These trends are clearly observed in Table 2 where the above correlations (Dataset1: *A* vs *amp*; Dataset2: *τ* vs *scale*) have the expected value of +1. In the other data sets, the minute changes in *A* and *τ* are prone to changes introduced by the addition of the AP template and the STD, leading to inconsistency.

The efficiencies of the convexity measurement algorithms were confirmed by evaluating their correlations with the ADP. As the ADP shares a strong positive correlation with the varied parameter in data sets 1 and 2, and a strong negative correlation in data sets 3 and 4 (see Section 2.2), a similar trend is expected with the measured convexity as well. The results obtained by different algorithms for each of the four data sets are given in Table 3. As predicted, the findings were identical to those in Table 2 except for a change in the sign for Datasets 3 and 4. Apart from testing the efficacy of the convexity quantification algorithms, this also establishes the fact that if the ADP is caused by an STD underlying the AP (Figure 3), the nature of the underlying STD could be studied by observing the correlation between the convexity of the AP foot and the ADP exhibited by the AP.

**Table 3:** Spearman’s rank correlation between the convexity measures obtained via the different quantification approaches and the extent of ADP.

Approach	Correlation with ADP in dataset			
	1	2	3	4
C_rad:min_	−1.00	1.00	−0.77	−0.86
C_rad:mean_	−1.00	0.99	−1.00	−0.82
C_rad:total_	−1.00	1.00	−1.00	−0.83
C_alt_	1.00	−1.00	−1.00	−0.83
C_exp:A_	1.00	1.00	0.77	−0.73
C_exp:_*_τ_*	−1.00	1.00	0.37	−0.21
C_area_	1.00	1.00	−1.00	−0.83
C_line_	1.00	1.00	−1.00	−0.83
C_20,0.6_	1.00	1.00	−1.00	−1.00

In summary, we conclude that the C_X,Y_ algorithm is recommended as the most suitable algorithm for quantifying the AP foot convexity, as it exhibited the best performance by yielding the expected correlations for all the test data sets.

### 3.3 Selection of Parameters X and Y in C_X,Y_

**Figure 9:**
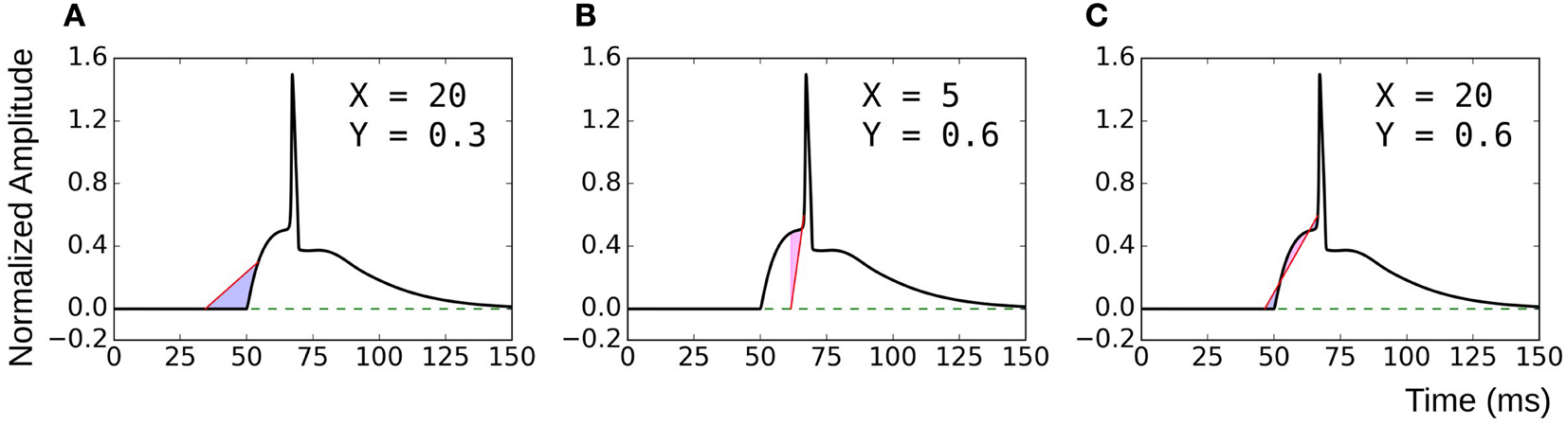
Choice of parameters X and Y. **(A)** and **(B)** are examples of inappropriate choices of X and Y. **(C)** covers both the entire height and width of the AP foot and is consequently a good choice of parameter values.

The proposed method of evaluating convexity, C_X,Y_, requires two parameters to be input, namely, X and Y. While evaluating C_X,Y_ on the test data sets, we had employed X = 20 ms and Y = 0.6. Here, we shall discuss the selection of values for these two parameters. Table 4 summarizes the mutual correlations for C_(X=20, Y=0.6)_, which has been demonstrated to be accurate, and other combinations of X and Y for each of the four data sets. We find that the first three cases (C_20,0.8_, C_30,0.6_, C_50,0.9_) are in agreement to C_20,0.6_ for all the data sets, whereas C_5,0.6_ and C_20,0.3_ differ. A simple rule of thumb in selecting values of X and Y is to set X to be larger than the maximum AP foot width and Y to be greater than the maximum AP foot height. This is illustrated in Figure 9 with examples of both appropriate and inappropriate choices of X and Y. In Figure 9A, the region of interest comprises only a part of the AP foot with the region above Y being excluded. Similarly, in Figure 9B, even though it covers the entire height of the AP foot, it does not span the entire foot duration. In such cases, as in (A) and (B), the convexity evaluation would be unreliable as variations in the foot shape outside the region of interest is ignored in the quantification. The choice of parameters in Figure 9C is such that the entire AP foot is taken into consideration. In the case of our test data, as we have prior knowledge of the variation in the AP profiles, it is trivial to set these values. For example, the maximum STD amplitude in our data sets is 0.5, and thus any value >0.5 is appropriate for Y. This rule is confirmed by the good correlation results for data sets 2, 3, and 4 under C_20,0.3_ (max *amp* = 0.2) as seen in Table 4, whereas it is found inappropriate for Dataset 1 (max *amp* = 0.5). It should also be noted that C_5,0.6_ appears a suitable choice for Dataset4, but not Dataset3. This is owing to the shorter range of *lat* parameter in Dataset4, resulting in smaller AP onset latencies and thereby shorter AP foot widths.

**Table 4:** Comparing correlations of C_X,Y_ for different combinations of X, Y parameter values with C_20,0.6_.

Data set	Spearman correlation of C_20,0.6_ with				
	C_20,0.8_	C_30,0.6_	C_50,0.9_	C_5,0.6_	C_20,0.3_
Dataset1	1.00	1.00	1.00	1.00	−0.61
Dataset2	1.00	1.00	1.00	0.98	1.00
Dataset3	1.00	1.00	1.00	0.73	1.00
Dataset4	1.00	1.00	1.00	1.00	1.00

As long as these conditions are satisfied, it is found that the choice of X and Y can be arbitrary. The magnitude of convexities evaluated would vary, based on the choice of X and Y, but the overall trends and relative values would remain consistent. When analyzing experimental signals, it might not be feasible to accurately identify these limits on X and Y. But as there is no upper bound on these parameters, it is advisable to overestimate their values. The strong correlation of C_20,0.6_ with C_50,0.9_ indicates that such large values provide equivalent results.

### 3.4 Analyzing Experimentally Recorded APs

**Figure 10:**
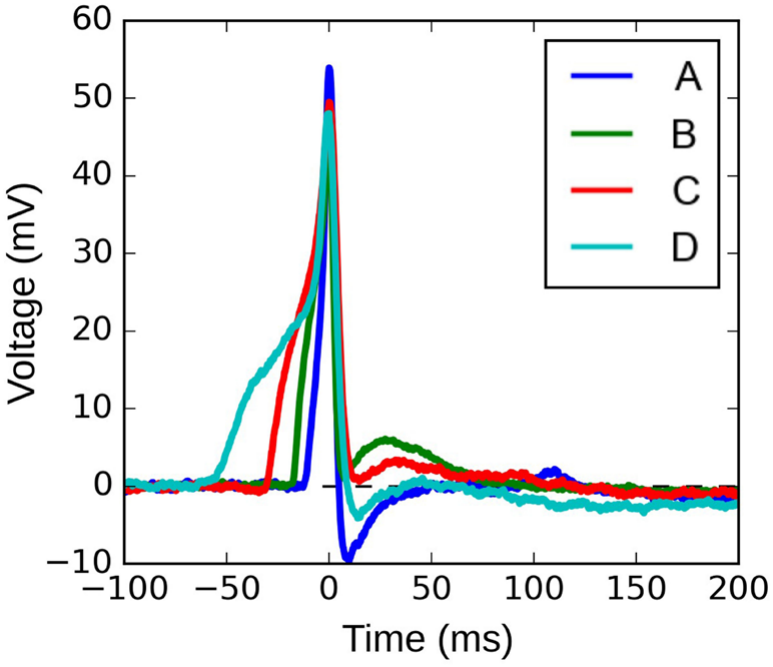
Four instances of experimentally recorded action potentials from the mouse detrusor. The peaks have been aligned at t = 0 ms.

**Figure 11:**
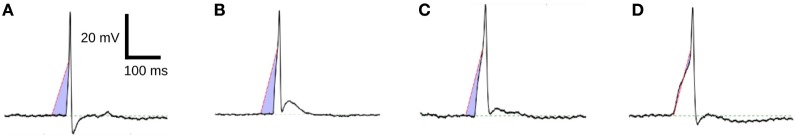
Quantification of convexities of experimentally recorded APs. The C_25,30_ convexity measures of signals **(A–D)** are −630.98, −525.68, −366.16, and −4.69, respectively.

Figure 10 shows four APs handpicked from a pool of intracellular recordings exhibiting varying extents of convexity in the AP foot, with the convexity increasing from A to D. These signals were analyzed using the C_X,Y_ approach, which was found to perform best amongst all the approaches tested. We set X = 50 ms and Y = 30 mV, in accordance with the rule of thumb described earlier. The quantification for these four APs are shown in Figure 11, with the measures being −630.98, −525.68, −366.16, and −4.69 for A, B, C, and D, respectively. It can be observed that the measured convexity trend matches the visually expected ordering of convexities for all the APs.

## 4 Discussion

Traditionally, APs are characterized mainly in terms of their height and width, and in case of bursting APs, also in terms of their spiking frequency. A feature of the AP that is often neglected, but can contain valuable information from which physiological inferences may be drawn, is the foot of the AP. To our knowledge, ours is the only study that focuses on quantifying the foot of APs, which describes their depolarization to threshold phase. As no established technique exists for this purpose, we explored some common mathematical paradigms that could be employed for such analysis. The various approaches were examined closely for their robustness when evaluating APs of a wide variety, and their shortcomings were identified. To overcome these inadequacies, we designed a new approach for quantifying the AP foot.

According to cable theory, when a passive signal, such as an STD, is observed at increasing distances from the site of origin, its amplitude is decreased, its time course is increased, and if an AP is elicited owing to that STD, the latency of the AP with respect to the STD is also increased. These variations are captured using the three parameters used to generate the test data set, namely, *amp, scale*, and *lat*. Even though these parameters are interconnected in the physiological scenario, certain meaningful inferences may be derived from individual variations of these parameters, as follows: *amp*—increase in amp indicates higher input resistance of the cell, or a larger quantity of neurotransmitter release, or proximity to varicosity; *scale*—wider time course of STD suggests propagation of STD from the site of neurotransmission, thereby offering an indication of distance from varicosity; *lat*—larger latency between the STD and AP can be an indicator of the higher threshold value of the cell to generate AP, and/or relatively large distance traveled by the STD-AP pair from the origin. As these physiological inferences, derived from the parameters that determine foot convexity, offer considerable insight into the intricacies of smooth muscle physiology, a quantification method that performs well for changes in each of the parameters is necessary.

The method to characterize action potential foot convexity thus forms an important tool on multiple fronts. These can broadly be classified into the following two categories.

*Analysis of Experimental Signals*: We are working toward the classification and decomposition of individual AP profiles to identify the “native” AP profile and the profile of the underlying synaptic potential. The latter is then compared against the collection of subthreshold synaptic potentials exhibited by the cell, to obtain inferences regarding the nature of innervation in the vicinity of the cell. Preliminary studies have shown that such comparisons show best results when employing the method presented here for quantifying the foot convexity.

*Comparison to Computational Model*: Simulations are run under a variety of syncytial configurations, such as variations in the size of the syncytium, density of innervation, gap junctional coupling, and so on (Appukuttan et al., 2015a, 2017a). These all translate to differences in action potential profiles exhibited across the syncytium. The features of these diverse action potential profiles would then be correlated with experimental data to obtain inferences of the cellular environment.

**Figure 12:**
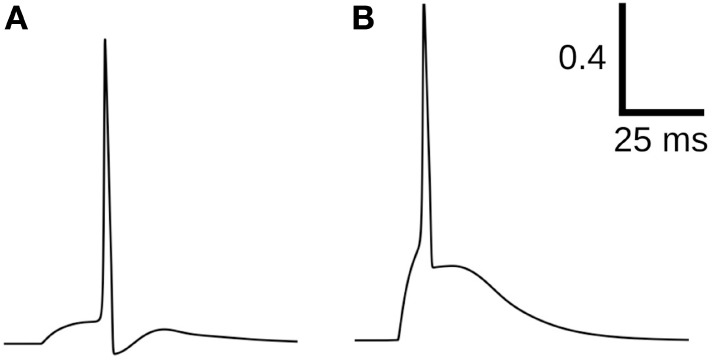
Comparison of convexity evaluation for C_20,0.6_ and C_line_. For **(A)**: C_line_ = 0.23, C_20,0.6_ = −4.78; for **(B)**: C_line_ = 0.16, C_20,0.6_ = −4.22.

Test data consisting of diverse AP shapes was essential for evaluating the various quantification approaches. These were carefully developed to ensure foreknowledge of convexity trends, which were later used as benchmarks for the evaluation of the approaches. All the approaches, excluding C_X,Y_, failed in evaluating the correlations for Dataset4, with varying levels of success for the other data sets. Dataset4 was the only data set containing an equitable proportion of APs with variations in their concave feet, along with APs having convex feet. This is particularly important in view of the fact that the bulk of the APs in an electrically coupled syncytium is likely to be composed of propagated APs having concave feet (Appukuttan et al., 2015a). In view of the results presented here, we conclude that C_X,Y_ is most efficient in quantifying the foot of action potentials. Figure 12 presents an example where C_line_ falters in quantifying the foot. Though it is evident that the AP in (A) has a less convex rise than that in (B), C_line_ assigns a higher convexity measure to the former (A: 0.23 vs B: 0.16), whereas C_20,0.6_ correctly assigns (B) a higher value (A: −4.78 vs B: −4.22).

Our approach is also shown to be effective in analyzing experimentally recorded signals, which are inherently noisy and offer greater diversity in profiles, thereby confirming the robustness of the approach. It is interesting to note from Figures 10 and 11 that the convexity vs ADP correlation seems to be in opposition to the proposed hypothesis (Padmakumar et al., 2012). The reason for this is beyond the scope of the current work, which purely aims at presenting an efficient approach for quantifying AP foot convexity. A detailed study directed at analyzing the variety of action potential profiles observed in mouse detrusor smooth muscle cells is currently underway. The first step in this study involves quantification of the various AP features, with the foot convexity being evaluated using the approach described here. It should be noted that erroneous quantification of the foot convexity could lead to incorrect interpretations regarding the biophysical environment. For example, a common issue with all the approaches, except C_X,Y_, is their inability to evaluate concavity of varying degrees in the AP foot, which could evolve an incorrect estimate of the size of syncytium.

One drawback of the C_X,Y_ technique is that it is not capable of differentiating between APs having convex and concave feet, as intuitively as in other techniques such as C_line_, C_alt_, C_rad_, or C_exp_. However, detection of convexity is not the objective here, but rather the quantification of the relative extents of convexity or concavity. In terms of the overall efficiency in quantification, C_X,Y_ surpasses all the other approaches presented here, displaying maximal accuracy with each data set, thereby making it the recommended method. Classification of APs into convex and concave categories, whenever required, could be achieved via clustering using the C_X,Y_ measures for each AP.

The profile of an action potential recorded in a cell is determined by an ensemble of intra-, extra-, and inter-cellular mechanisms. These include the composition of ionic channels, cytosolic calcium dynamics, synaptic input, and also, in the case of syncytial tissues, factors such as gap junctional coupling and the arrangement of cells in bundles. Certain pathologies of the bladder, such as detrusor overactivity, are reported to have a myogenic origin (Brading, 1997), while some others have a neurogenic basis (de Groat, 1997). In the former, changes are believed to take place in the SMCs and/or their interaction with other SMCs in the syncytium (Fry et al., 2002), whereas the latter involves changes in the pattern and/or density of parasympathetic innervation. Changes in any of these factors will affect the AP profile exhibited by the cell. For example, an increase in the frequency of APs with a convex foot could suggest an increase in the density of parasympathetic innervation, while a higher proportion of APs with concave feet could be indicative of stronger coupling and the formation of syncytia of larger sizes, e.g., those in which APs propagate over such distances that the convex foot is lost. With intracellular recordings, therefore, an analysis of the AP shapes holds potential for understanding the cellular environment within the detrusor smooth muscle, and may help evaluate biophysical changes that occur in pathology.

Intracellular recordings cannot directly be used for clinical investigations, because of the difficulties inherent in *in vivo* intracellular investigations. However, insights obtained from analysis of intracellular APs can be extended and combined with other types of recordings that are feasible in clinical or para-clinical settings. For instance, in extracellular or tissue-surface recordings which are performed clinically, spikes recorded extracellularly are either biphasic in shape (negative–positive in polarity) when recorded at or very close to their sites of origin, or triphasic in shape (positive–negative–positive in polarity) when recorded at a distance following propagation from the source (Jack et al., 1975; Obien et al., 2015; Lewandowska et al., 2015; Appukuttan et al., 2017b). The curvature or the degree of convexity present in the different phases can shed light on underlying biophysical mechanisms, akin to the case of intracellular recordings. In the case of muscles, a precise understanding of whole-organ biophysics based on such techniques can be of considerable clinical use, as has been demonstrated in the case of skeletal muscle, helping to distinguish between, for example, disorders that affect primarily the neuromuscular junction as against those that affect primarily the propagation of spikes or excitation-contraction coupling (Daube, 2002). Profiling of the electrical activity in excitable cells has proven valuable, in the past, toward interpreting the nature of autonomic neurotransmission. For example, the differentiation of the rising phase of excitatory junction potentials (EJPs) in the vas deferens and their subsequent analysis identified the presence of discrete events (DEs) representing the release of neurotransmitter from a single varicosity (Blakeley and Cunnane, 1979). These DEs have enabled fingerprinting of different neurotransmitter release sites around the recorded cell (Cunnane and Stjärne, 1984). Similarly, spontaneous discrete events (SDEs) were obtained by differentiation of the rising phase of spontaneous excitatory junction potentials (sEJPs). Subsequent analysis found that when a particular release site is activated, the resultant evoked DE corresponded precisely with a particular SDE recorded from that cell (Cunnane and Stjärne, 1984; Manchanda, 1995). Further studies found that the currents underlying sEJPs and EJPs shared similar amplitude distributions, and template matching showed that certain spontaneous excitatory junction currents (sEJCs) and excitatory junction currents (EJCs) were identical in their amplitude and time course (Brock and Cunnane, 1988). These findings provided considerable insight into the biophysical environment of the recorded cells, such as the properties of quantal neurotransmitter release in sympathetic nerve terminals, and the probability of stimulation-evoked release. Extensions of the work reported here could result in similar analytical approaches to detrusor smooth muscle that could lend themselves eventually to clinical electrodiagnostics.

## Ethics Statement

The Institutional Animal Care and Use Committee (IACUC) covering the Department of Pharmacology, University of Oxford, approved and had oversight of all animal experiments. All experiments were carried out during or before 2009, and were hence approved under Animals (Scientific Procedures) Act 1986, but are also consistent with both UK Animals (Scientific Procedures) Act (2013) and European Communities Council Directive 2010/63/EU.

## Author Contributions

SA designed the computational studies, performed the simulations, and wrote the first draft of the manuscript. MP designed the analysis of experimental data, carried out the statistical analysis, and contributed in writing the manuscript. KB performed the electrophysiological recordings and helped revise the manuscript. RM contributed toward the interpretation of the results and in revising the manuscript.

## Conflict of Interest Statement

The authors declare that the research was conducted in the absence of any commercial or financial relationships that could be construed as a potential conflict of interest.

## References

[B1] Appukuttan S. Brain K. Manchanda R. (2015a). Syncytial basis for diversity in spike shapes and their propagation in detrusor smooth muscle. Proc. Comput. Sci. 51, 785–794. 10.1016/j.procs.2015.05.199

[B2] Appukuttan S. Brain K. L. Manchanda R. (2015b). A computational model of urinary bladder smooth muscle syncytium. J. Comput. Neurosci. 38, 167–187. 10.1007/s10827-014-0532-6 25292316

[B3] Appukuttan S. Brain K. Manchanda R. (2017a). Investigation of action potential propagation in a syncytium. Biomed. Res. J. 4(1), 102–115.

[B4] Appukuttan S. Brain K. L. Manchanda R. (2017b). Modeling extracellular fields for a three-dimensional network of cells using neuron. J. Neurosci. Methods 290, 27–38. 10.1016/j.jneumeth.2017.07.005 28705695

[B5] Bean B. P. (2007). The action potential in mammalian central neurons. Nat. Rev. Neurosci. 8, 451–465. 10.1038/nrn2148 17514198

[B6] Bennett M. Gibson W. Poznanski R. (1993). Extracellular current flow and potential during quantal transmission from varicosities in a smooth muscle syncytium. Philos. Trans. R. Soc. Lond. B Biol. Sci. 342, 89–99. 10.1098/rstb.1993.0140 7904356

[B7] Blakeley A. Cunnane T. (1979). The packeted release of transmitter from the sympathetic nerves of the guinea-pig vas deferens: an electrophysiological study. J. Physiol. 296, 85. 10.1113/jphysiol.1979.sp012992 231103 PMC1279065

[B8] Brading A. F. (1997). A myogenic basis for the overactive bladder. Urology 50, 57–67. 10.1016/S0090-4295(97)00591-8 9426752

[B9] Brock J. Cunnane T. (1988). Electrical activity at the sympathetic neuroeffector junction in the guinea-pig vas deferens. J. Physiol. 399, 607. 10.1113/jphysiol.1988.sp017099 2900334 PMC1191683

[B10] Bywater R. Taylor G. (1980). The passive membrane properties and excitatory junction potentials of the guinea pig deferens. J. Physiol. 300, 303–316. 10.1113/jphysiol.1980.sp013163 7381788 PMC1279356

[B11] Cunnane T. Stjärne L. (1984). Transmitter secretion from individual varicosities of guinea-pig and mouse vas deferens: highly intermittent and monoquantal. Neuroscience 13, 1–20. 10.1016/0306-4522(84)90255-0 6149492

[B12] Daube J. R. (2002). Assessing the motor unit with needle electromyography. Contemp. Neurol. Ser. 66, 293–323. 10.1093/med/9780195385113.003.0026

[B13] de Groat W. C. (1997). A neurologic basis for the overactive bladder. Urology 50, 36–52. 10.1016/S0090-4295(97)00587-6 9426749

[B14] Fatt P. Katz B. (1951). An analysis of the end-plate potential recorded with an intra-cellular electrode. J. Physiol. 115, 320–370. 10.1113/jphysiol.1951.sp004675 14898516 PMC1392060

[B15] Fry C. H. Skennerton D. Wood D. Wu C. (2002). The cellular basis of contraction in human detrusor smooth muscle from patients with stable and unstable bladders. Urology 59, 3–12. 10.1016/S0090-4295(01)01632-6 12007516

[B16] Hashitani H. Brading A. F. (2003). Ionic basis for the regulation of spontaneous excitation in detrusor smooth muscle cells of the guinea-pig urinary bladder. Br. J. Pharmacol. 140, 159–169. 10.1038/sj.bjp.0705320 12967945 PMC1573995

[B17] Hines M. Carnevale N. T. (2001). NEURON: a tool for neuroscientists. Neuroscientist 7, 123–135. 10.1177/107385840100700207 11496923

[B18] Hirst G. Neild T. (1978). An analysis of excitatory junctional potentials recorded from arterioles. J. Physiol. 280, 87. 10.1113/jphysiol.1978.sp012374 690942 PMC1282649

[B19] Jack J. J. Noble D. Tsien R. W. (1975). Nonlinear Cable Theory: Conduction. Oxford: Clarendon Press.

[B20] Kreyszig E. (1991). Principal Normal, Curvature, Osculating Circle. Differential Geometry. New York: Dover, 34–36.

[B21] Lewandowska M. K. Bakkum D. J. Rompani S. B. Hierlemann A. (2015). Recording large extracellular spikes in microchannels along many axonal sites from individual neurons. PLoS ONE 10:e0118514. 10.1371/journal.pone.0118514 25734567 PMC4348166

[B22] Magee J. C. Carruth M. (1999). Dendritic voltage-gated ion channels regulate the action potential firing mode of hippocampal CA1 pyramidal neurons. J. Neurophysiol. 82, 1895–1901. 10515978 10.1152/jn.1999.82.4.1895

[B23] Manchanda R. (1995). Membrane current and potential change during neurotransmission in smooth muscle. Curr. Sci. 69, 140–150.

[B24] Meng E. Young J. S. Brading A. F. (2008). Spontaneous activity of mouse detrusor smooth muscle and the effects of the urothelium. Neurourol. Urodyn. 27, 79–87. 10.1002/nau.20456 17487871

[B25] Obien M. E. J. Deligkaris K. Bullmann T. Bakkum D. J. Frey U. (2015). Revealing neuronal function through microelectrode array recordings. Front. Neurosci. 8, 423 10.3389/fnins.2014.00423 25610364 PMC4285113

[B26] Padmakumar M. Bhuvaneshwari K. Manchanda R. (2012). “Classification and analysis of electrical signals in urinary bladder smooth muscle using a modified vector quantization technique,” in Signal Processing and Communications (SPCOM), 2012 International Conference On (Bangalore, India: IEEE), 1–5. 10.1109/SPCOM.2012.6290248

[B27] Padmakumar M. Brain K. Manchanda R. (2016). “Feature detection and classification of action potentials from detrusor smooth muscle cells,” in International Conference on Systems in Medicine and Biology (ICSMB) (Kharagpur, India: IEEE). 10.1109/ICSMB.2016.7915109

[B28] Purves R. (1976). Current flow and potential in a three-dimensional syncytium. J. Theor. Biol. 60, 147–162. 10.1016/0022-5193(76)90160-0 957706

[B29] Young J. S. Meng E. Cunnane T. C. Brain K. L. (2008). Spontaneous purinergic neurotransmission in the mouse urinary bladder. J. Physiol. 586, 5743–5755. 10.1113/jphysiol.2008.162040 18936079 PMC2655397

